# Supplementary figures and images for: Distribution and transmission of M. tuberculosis in a high-HIV prevalence city in Malawi: A genomic and spatial analysis
Source: PLOS Glob Public Health. 2025 Apr 2;5(4):e0004040. doi: 10.1371/journal.pgph.0004040 (PMC11964229; doi:10.1371/journal.pgph.0004040)

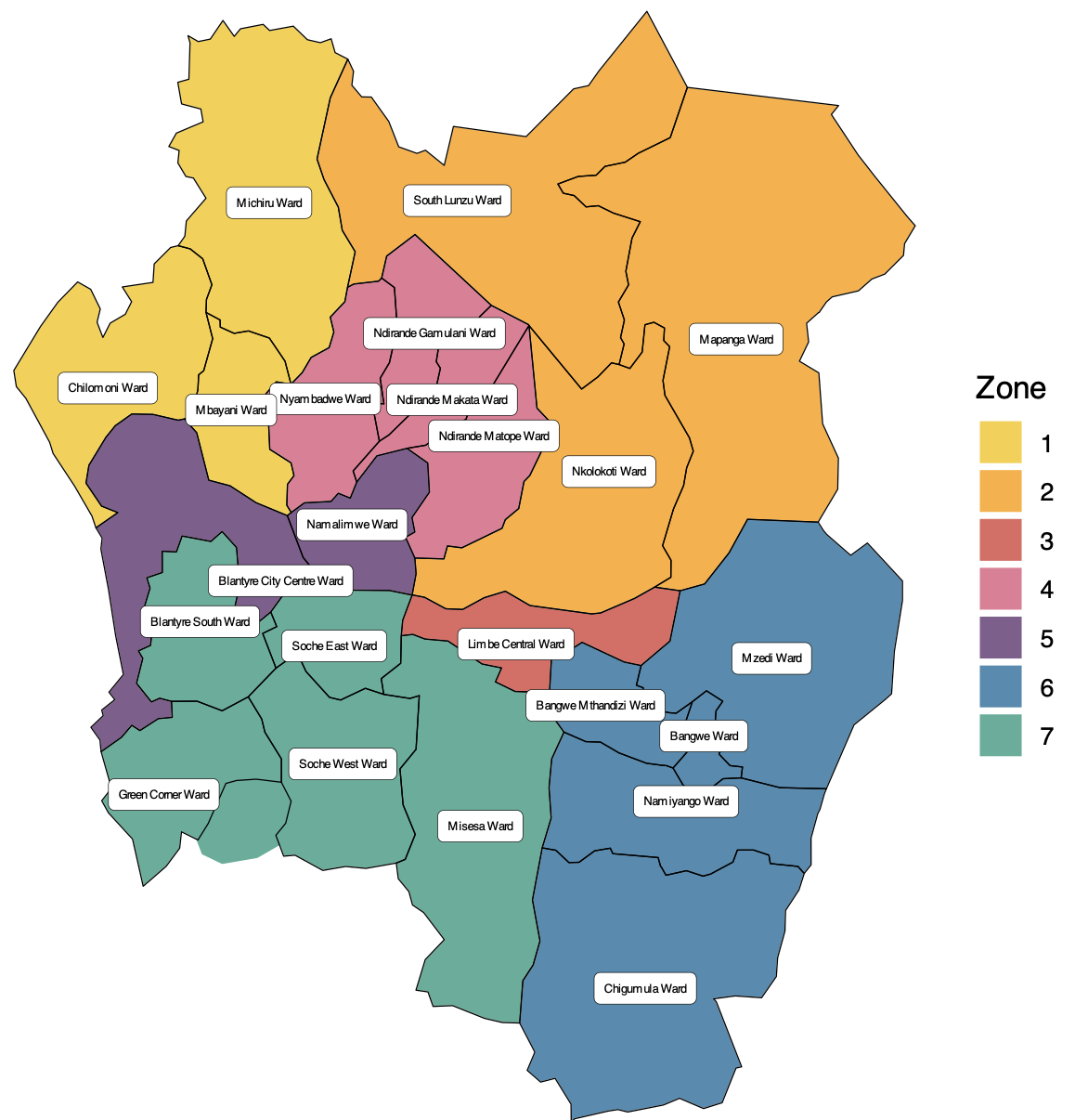

Supplement: S1 Fig — Zone 7 has been expanded to include a small area outside the city boundary with a high TB notification rate (unlabeled ward between Green Corner Ward and Soche West Ward). Zones drawn by authors based on existing administrative boundaries. Base map citation: Humanitarian Data Exchange, Accessed May 1 2023, https://data.humdata.org/dataset/cod-ab-mwi CC-BY-IGO. (TIFF) [file pgph.0004040.s001.tiff]

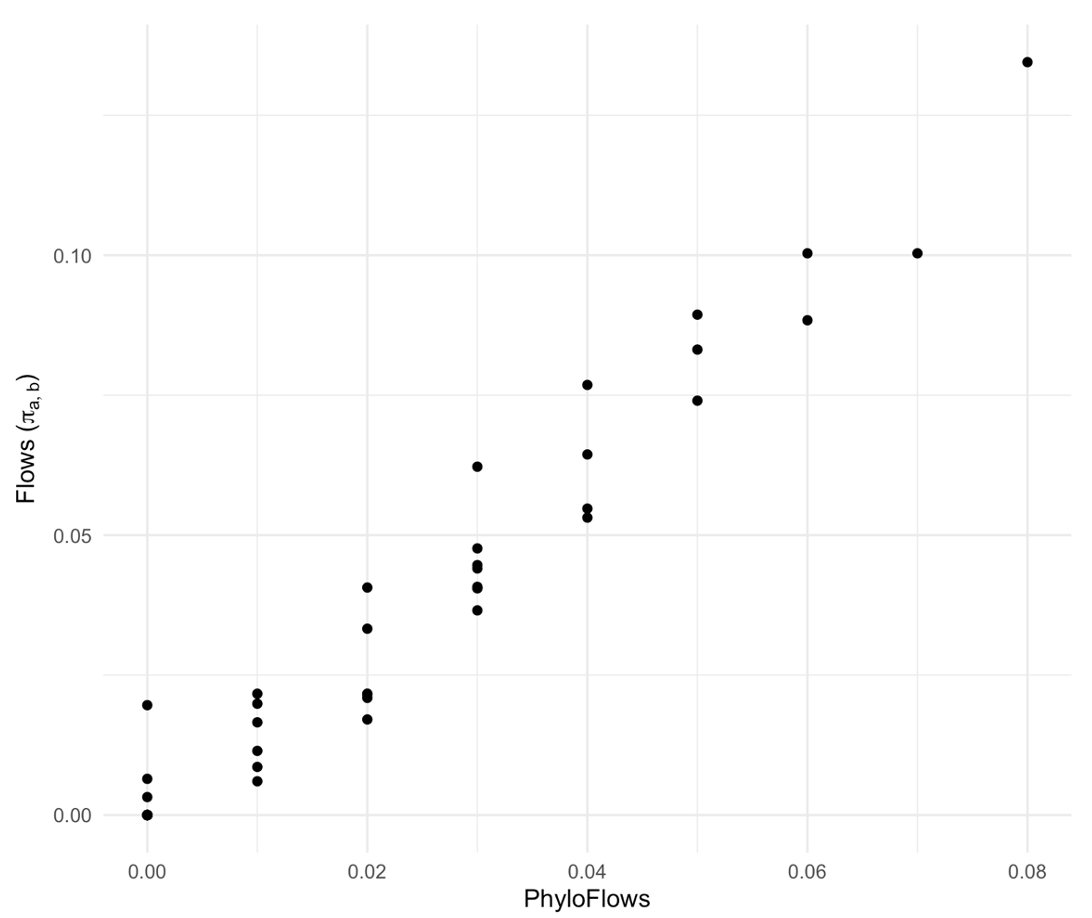

Supplement: S2 Fig — (TIFF) [file pgph.0004040.s002.tiff]

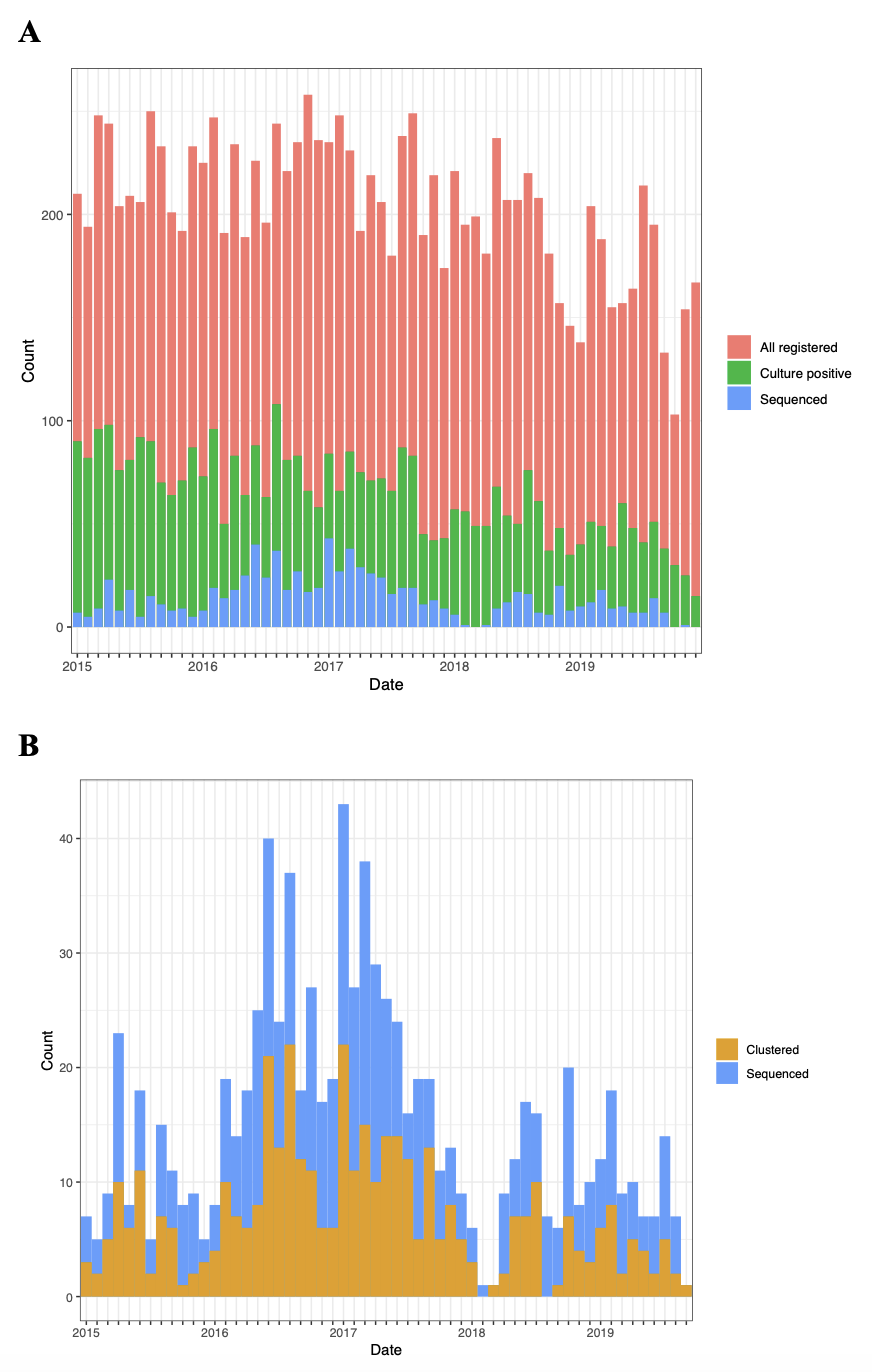

Supplement: S3 Fig — (TIFF) [file pgph.0004040.s003.tiff]

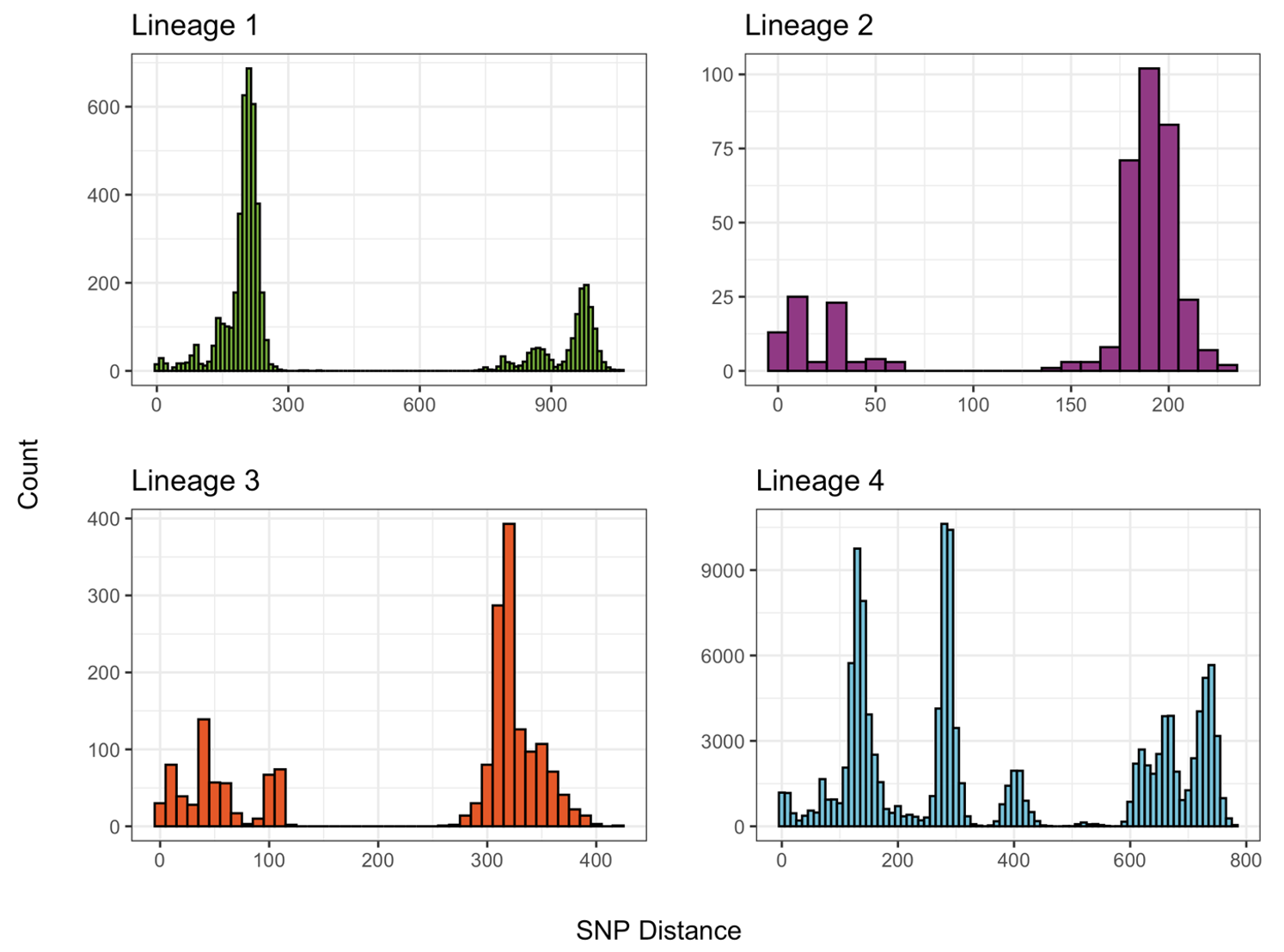

Supplement: S4 Fig — (TIFF) [file pgph.0004040.s004.tiff]

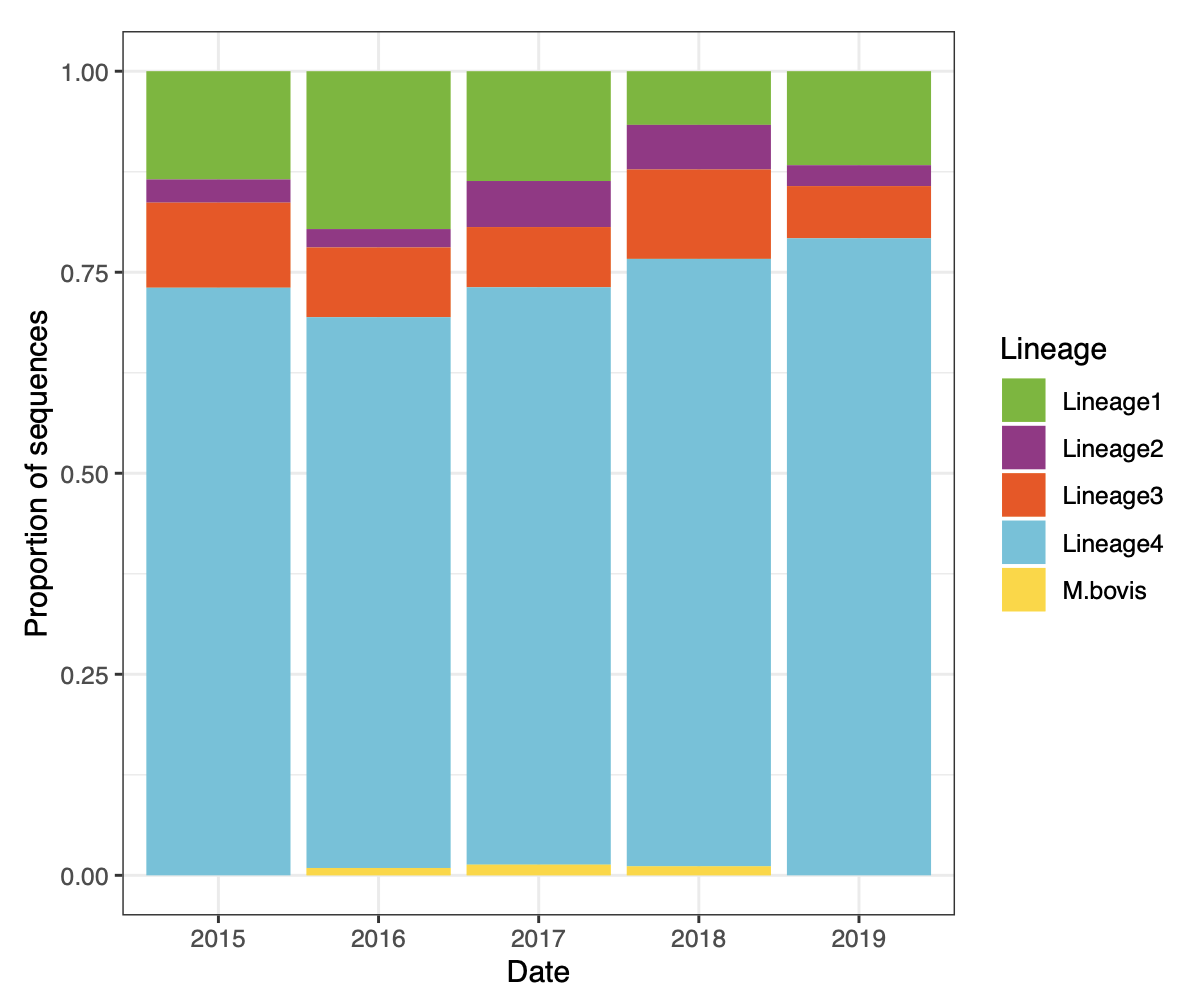

Supplement: S5 Fig — (TIFF) [file pgph.0004040.s005.tiff]

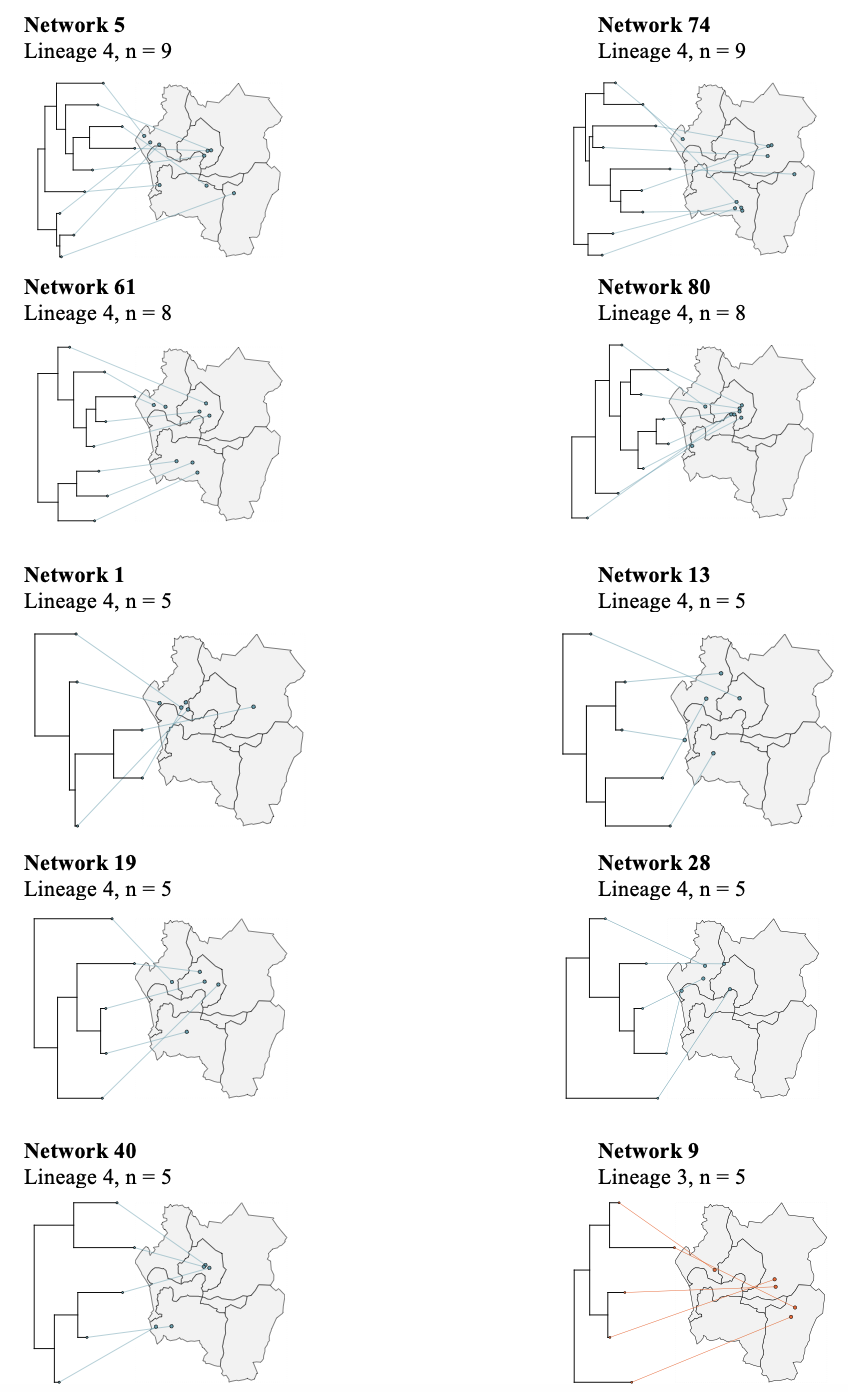

Supplement: S6 Fig — (TIFF) [file pgph.0004040.s006.tiff]
