# Supplementary material for: Distribution and transmission of M. tuberculosis in a high-HIV prevalence city in Malawi: A genomic and spatial analysis
Source: PLOS Glob Public Health. 2025 Apr 2;5(4):e0004040. doi: 10.1371/journal.pgph.0004040 (PMC11964229; doi:10.1371/journal.pgph.0004040)
Supplement: Attachment — Submitted filename: ResponseLetter.docx [file pgph.0004040.s015.docx]

**Reviewer #1:** Use of whole genome sequencing to identify transmission within Blantyre, Malawi.

Strengths of the study 
1. This is a very sophisticated study utilizing multiple sources of information and complex analysis methods to examine transmission, spatial foci, trend of transmission and transmission networks within a city in sub-Saharan Africa. The methods are strong and well described. 
2. ePAL - the availability of an electronic system that prospectively captures clinical data and geolocation on all patients diagnosed with active TB within Blantyre is a major strength.
3. Geographic data - the fact that they're able to localize individuals to their home residence and have detailed information on census data as well as HIV data with resolution of 500 m2 (or less) is another strength.
4. The use of sophisticated analysis techniques that are all available in R package, I find a particular strength since R is freely available hence the methods are more accessible to others, more generalizable and reproducible.
 
Weaknesses/Comments
1. Attrition: There were a little over 11,000 individuals with TB disease notified in the study period, of whom only 3856 had positive cultures, with WGS and patient data available for only 1009, and finally WGS was successful for only 707. Hence this analysis is performed on about 6% of all TB disease episodes, representing a rather small fraction of the total. Potential for selection bias seems important and yet I could not find a table comparing characteristics of the 11,000 to any of the subgroups, such as the 3856 who were culture positive or the final 707 included in the analysis.

This analysis was largely focused on individuals with culture positive disease. Common to many TB programmes around the world, a substantial fraction of people notified with TB do not have microbiologically-confirmed disease, usually because they cannot produce a sputum sample, or are treated on clinical suspicion of TB. A strength of this analysis is that we only included participants with culture-confirmed TB. We make mention of the total notification rate in order to provide additional context about the burden of TB in Blantyre. There were no systematic differences between the individuals who did or did not receive culture:

We have added additional information to help readers assess the possibility of selection bias by adding a new Table 1 which compares the characteristics of individuals for whom we have sequence data (n = 717) and the population of individuals with positive cultures and no sequencing data (n = 3148). As above, we found no systematic differences between the two groups:

2. Although there is very detailed information on location of transmission, probably the most important single piece of information that I was looking for is whether transmission was within households or not. I would have thought this information would potentially be available in the ePAL. If household contact investigations were routinely done, and if those detected with TB disease as a result of household contact investigation were notified as such within the electronic reporting system, then should be analyzed and reported here. From a programmatic and scientific point of view, the proportion of all transmission that can be attributed to household, versus non household or community level transmission is a very basic piece of information that would be very valuable to understand.

We did not have information about whether individuals in our study lived in the same household. This is because it is extremely challenging to correctly classify household membership where there is no municipal address system. However, we investigated potential household transmission events in two ways. First, we identified individuals who reported having a household contact with TB. Second, we identified plausible shared households based on the proximity of home GPS coordinates. We did not find genomic evidence of transmission for individuals in either group, though we note that this may be related to the coverage of whole genome sequencing. We have added a new paragraph to the result describing these findings:

“We also attempted to identify household transmission events. In total, 270 individuals (2.2% of the TB notifications over the study period) reported a household contact with TB. The final sample dataset included 44 of these individuals (6.1% of the dataset); among these individuals, 21 (47.7%) belonged to a transmission network. Individuals who reported having a household contact with TB were not more likely to belong to a transmission network than individuals who did not report a household contact (two-sample proportion test, p = 0.36). In addition, while 19 individuals could plausibly belong to the same household as another individual in the final sample dataset (i.e. home GPS coordinates were within 50 meters), none of these plausible household pairs belonged to the same transmission network.”

3. Guide for programs: Along these lines, do the authors consider this potentially useful to guide programs to identify hotspots for transmission where TB control efforts can be concentrated such as, for example, active case finding efforts targeted to hot spots for transmission. The finding that clustering or evidence of transmission appears to be associated with attending the same TB clinic, for example, seems like a very relevant piece of information but ideally we'd have a bit more information about this in order to have some evidence that might guide TB program practice.

Thank you for this feedback. Our findings reveal a complex epidemic involving both local transmission within neighborhoods, and cross-city transmission. These patterns were not evident before the study and translate into clear policy recommendations: improving access to tuberculosis services through primary clinics citywide, and that supplementing these general improvements with targeted active case finding. We also believe that these findings provide necessary background information to be used in the context of transmission models to better understand the potential impact of spatially targeted case finding interventions, and to assess whether such interventions are likely to generate sufficient health benefits to justify their costs. In order to better reflect this complexity, we have made several edits to the Discussion section. We have removed the initial recommendations in the first paragraph of the Discussion in favor of a more nuanced paragraph at the end of Discussion; this takes the place of our original concluding paragraph:

“Whole genome sequencing data provide information about local transmission and help to identify geographic areas where transmission is highest. Focused interventions to interrupt transmission could be effective at reducing incidence in these areas, and previous models have suggested that interventions targeted to a single transmission focus may have broader benefits to reducing incidence in an entire city^(6)^. However, given the interconnectedness of transmission in Blantyre, the degree to which targeting interventions to small numbers of geographic foci will have these positive ‘spill-over’ effects in unclear. Transmission models calibrated to the spatial transmission patterns estimated in this study provide an attractive next step for estimating the potential health benefits of targeting interventions to transmission foci. In the absence of such estimates, our results suggest that TB control efforts should focus on improving access to tuberculosis services through primary clinics citywide, and that supplementing these general improvements with targeted active case finding should be considered as a secondary tactic.”

4. Tables - there is very heavy emphasis on figures. These are interesting, but I would like to see some of the information summarized in tabular form, for example basic characteristics of the individuals who were diagnosed with TB disease in this period, and those who are included in this analysis would be helpful, similarly, the characteristics of those within clusters and not within clusters, those in clusters of two vs larger clusters, etc.

We have added a new table (Table 1) to the main text to show characteristics of individuals in the study.

Overall, I find the paper very sophisticated, the analyses complex, and methods were excellent. However I don't find any clear and practical messages for the readers; I wonder if a general medical journal like PLOS Medicine is the right place for this? Have the authors or editors considered a more specialized journal for this type of work? 

We appreciate your positive feedback on the analyses and methods. We have now expanded the discussion to provide a more policy-relevant focus.

**Reviewer #2:** The manuscript entitled "Distribution and transmission of M. tuberculosis in a high-HIV prevalence city in Malawi: a genomic and spatial analysis" by M. Chitwood and colleagues entails a considerable effort in gathering epidemiological and M. tuberculosis sequence data for a major urban centre in Malawi. The authors have taken advantage of an electronic participant locator platform which enabled comprehensive data collection for each participant and were able to have paired clinical isolates and obtain sequence data for 717 isolates. The main goal was to infer on transmission patterns across multiple geographical zones and for this purpose the authors implemented a series of transmission modelling approaches that harness genomic data to further integrate the genetic diversity and proximity evidenced from the genomic data. However, given the dimension of the sample size and its timespan, I personally find that the study has fallen short of its potential. My main concerns are:

1.      Regarding novelty and impact, it is still unclear how the results presented will assist and inform public health authorities to come up with more effective programmatic control procedures. At some points the rationale on this topic is confusing, particularly in the first paragraph of the discussion where the authors propose that improved access to healthcare is an important factor given that transmission occurs between zones. This statement is almost always true, and I struggle to find a connection between the data obtained and this conclusion. The same for active case finding. The authors must make it more clear how the identification of enhanced transmission between zones and the identification of hotspots can make a difference in terms of programmatic management and transmission control;

Thank you for your feedback. We have added a new paragraph to the Discussion section. Reviewer 1 raised a similar concern. Please see our response to Reviewer 1:

Our findings reveal a complex epidemic involving both local transmission within neighborhoods, and cross city transmission. These patterns were not evident before the study and translate into clear policy recommendations: improving access to tuberculosis services through primary clinics citywide, and that supplementing these general improvements with targeted active case finding. We also believe that these findings provide necessary background information to be used in the context of transmission models to better understand the potential impact of spatially targeted case finding interventions, and to assess whether such interventions are likely to generate sufficient health benefits to justify their costs. In order to better reflect this complexity, we have made several edits to the Discussion section. We have removed the initial recommendations in the first paragraph of the Discussion in favor of a more nuanced paragraph at the end of Discussion; this takes the place of our original concluding paragraph:

“Whole genome sequencing data provide information about local transmission and help to identify geographic areas where transmission is highest. Focused interventions to interrupt transmission could be effective at reducing incidence in these areas, and previous models have suggested that interventions targeted to a single transmission focus may have broader benefits to reducing incidence in an entire city^(6)^. However, given the interconnectedness of transmission in Blantyre, the degree to which targeting interventions to small numbers of geographic foci will have these positive ‘spill-over’ effects in unclear. Transmission models calibrated to the spatial transmission patterns estimated in this study provide an attractive next step for estimating the potential health benefits of targeting interventions to transmission foci. In the absence of such estimates, our results suggest that TB control efforts should focus on improving access to tuberculosis services through primary clinics citywide, and that supplementing these general improvements with targeted active case finding should be considered as a secondary tactic.”

2.      A different aspect concerns the robustness of the data and this plays a pivotal role for the entire study. The authors do recognize that the transmission modelling effort usually requires a more dense sampling strategy and since the mathematical modelling of transmission will be an approximation, special care must be taken to ensure that the data is valid and is further supported by an orthogonal approach. As it stands the manuscript misses this and specific points can be pointed out: 

i)      the population structure of the dataset is completely overlooked, few lines mention the lineages found, which are in fact annotated onto the tree, but for me this is insufficient. A more detailed analysis of the population structure and sub-lineages should have been detailed;

Thank you for your feedback. We have included a new supplementary figure showing the distribution of pairwise distances by major lineage. We have also added new text to the Results section about the diversity within lineages:

“The diversity of strains differed markedly by lineage; lineages 1 and 4 had larger pairwise SNP distances, on average, as compared to lineages 2 and 3 (Supplemental Figure 3)”

ii)     are the transmission networks corroborated by a SNP-based cluster definition? Do the isolates in these transmission networks form monophyletic branches in the phylogenetic tree? No information is provided regarding this and it would help the reader to better rationalize over the findings that are here reported;

The transmission networks do form monophyletic clades in the phylogenetic tree. We have added a sentence to the results to indicate that these networks are corroborated by SNP distances:

“The average pairwise SNP distance within a network was 4.5 (IQR: 1, 7).”

We agree that it would be useful to visualize these networks on the phylogeny. We have updated Figure 2 to include a heatmap indicating which taxa belong to transmission networks; we also highlight the taxa belonging to the three largest transmission networks in the study.

iii)    drug resistance is briefly mentioned and, for one, I would expect more given the country's overall MDR/RR-TB rate during the study period. Yet, only 6 isolates were RR/MDR-TB. Is there a possible bias? This aspect isn't discussed nor is the eventual distribution of these drug resistant isolates across transmission networks or its positioning in the tree.

During the study period, the rate of RR/MDR in Malawi was very low at 0.75% (7.5 per 1000 cases) for new cases and 6.4% in relapse cases (see: <https://pmc.ncbi.nlm.nih.gov/articles/PMC8542254/>). A whole genome sequencing based analysis in Karonga, Malawi found that RR-TB was present in 0.8% of cases (see: <https://www.microbiologyresearch.org/content/journal/mgen/10.1099/mgen.0.000361>). Our finding of RR-TB in 0.8% of sequenced cases is consistent with what is known about drug resistance prevalence in Malawi during this time period. The low prevalence of drug resistant TB in Malawi has been attributed to the strong national TB programme’s public health approach to treatment and care.

Of the 6 isolates with RR/MDR-TB, three belong to a transmission network. Of these three, two belong to the same transmission network of size two; the third belongs to a separate network also of size two. Our analyses suggest that the vast majority of transmission in Blantyre is transmission of drug susceptible *Mtb*.

3.       It is not mentioned how was lineage or drug resistance determined. Which software was used and is the drug susceptibility data based on genotypic prediction or was it determined phenotypically?

Lineage prediction and drug resistance profiling were carried out *in silic*o using TB Profiler. This is described in the final sentence of the ‘Laboratory regrowth, DNA isolation, and whole genome sequencing’ subsection of the Methods.

4.      It is also unclear how do the authors conclude that "high levels of population mixing play a role in sustaining Blantyre's tuberculosis epidemic". As it is written, the authors convey a very strict notion of zones that are likely to have no relation in daily populational movements across a common urban centre. Did the authors collect data on the location of workplaces? This information would be essential for determining the daily patterns of population movement and how it occurs between zones.

Zones were determined in collaboration with the National TB Programme and members of the research team in Malawi, and they were defined based on existing administrative boundaries. We agree that additional information about daily patterns of movement would enable us to speak more concretely about the reasons for transmission that occurs among individuals who do not reside near each other. We have softened the language of this sentence to convey that we are speculating about a possible reason for the high levels of inter-zone transmission events:

“…high levels of population mixing may play a role in sustaining Blantyre’s tuberculosis epidemic.”

Minor concerns:

-       In the Methods section please mention the tree prior model used in BEAST.

We have included this detail; we used a constant coalescent population tree prior.

-       In the discussion, the authors mention that residential proximity is an important predictor and mention Figure 5A. I can't find this observation in Figure 5A. The only covariates that appear to be related are the "Same TB clinic" and "Same HIV clinic" but even these are contradictory.

We had opted to report on the effect of distance but exclude it from the figure because we believed displaying two distance effects (above and below 6km) may be confusing. Because the exclusion appears to have caused more confusion, we have added these predictors back into figure 5A:

**Reviewer #3:** Thank you for the opportunity to review this paper. This paper has many strengths, including the use of population-based TB surveillance data from a high HIV-prevalent country. I applaud the authors for this tremendous effort. I find several flaws in the article. These include:

-       In the Abstract, the objective sentence ("We investigated genomic diversity…" seems overly broad and not entirely consistent with what is in the main text. The objectives in the main text is also somewhat vague. I recommend describing more specifically what the study aimed to achieve, which will allow the reader to understand why the various analyses were done.

We have removed the reference to genomic diversity from the abstract to refocus on our goal of describing transmission patterns. We have also added a new sentence in the Introduction section to more clearly state the objective of the study:

“The aim of this study was to describe patterns of TB transmission in an endemic setting using high-resolution surveillance and WGS data.”

-       Abstract states, "We found that 56% of individuals with sequenced isolates had a probable direct transmission link to at least one other individual in the study." Please confirm this statement as I am unable to find this in the Results. The only 56% I see is 393 isolates (56%) that belonged to one of 130 transmission networks inferred with TransPhylo and had an associated GPS coordinate." This is different than having a probable direct transmission link as it includes networks of observed and unobserved individuals with TB.

Thank you for catching this oversight. We have removed the word ‘direct’. As you rightly point out, there are unsampled intermediates in these transmission chains.

-       From the Results, it looks like 144 isolates with mixed infections were excluded from the analysis. In the Limitations section, please describe the possible implications of removing this data on study conclusions.

We have included new text in the Discussion section discussing our rationale for removing these strains and the impact this omission has on sample size.

“Additionally, 144 sequences were excluded due to evidence of polyclonal infections, which reduced the fraction of cases which could be included in this study because of the challenge in including these types of complex samples in phylogenetic reconstruction and transmission analysis."

-       For the spatial foci analysis, was the underlying population density accounted for in the analysis? If not, is it possible that the foci are determined largely by higher population density?

We do not need to account for population density when identifying transmission foci using the distance-based mapping approach. This approach allows us to identify areas where the fraction of cases belonging to a specific network (‘cases’) compared to all other sequenced cases (‘controls’) is higher than would be expected in a null scenario where the ‘case’ and ‘control’ classifications are randomly assigned. The foci are not a function of population density.

-       The spatial foci were based on home location data. Please comment on the potential limitations of not including data on other important activity spaces, including school and work.

We have added new text to the Discussion section to highlight this limitation of our spatial analyses:

“We note that these spatial foci were identified based only on the home location of individuals with TB. While we investigate the potential role of clinics in the dyadic regression analysis, we did not have data about other locations where transmission may occur, such as schools, workplaces, or other congregate settings.”

-       It is unclear what the purpose of the pairwise regression analysis is. Please provide a justification and aims for doing this analysis.

-       It is unclear to me what the outcome variable is for the pairwise regression analysis. The Methods seems to suggest "genetic relatedness of pairs of cases" as the outcome while the Results seems to show "being in a transmission network" as the outcome. Please clarify this and provide details about the format of the outcome variable (e.g. numerical variable of SNP differences?).
-       For the regression analysis of large transmission clusters, the measure of association is the risk ratio, which implies that the outcome is binary. However, the outcome is described in the figure as having "fewer SNP differences". Please describe more clearly how the outcome is formatted.  
-       For the regression analysis of large transmission clusters, the number of observations are 25, 17, and 15. These numbers seem much too small to accommodate 10 predictor variables. Please justify fitting such models with sample sizes that seem to be too small for them.

We have made several changes to the Methods and Results sections to clarify the purpose, design, and outcomes of the regression analyses. Briefly, the goal of these analyses is to estimate the association of specific factors with putative transmission. The unit of analysis is a pair of individuals.

We conduct two separate regression analyses. The first is a logistic regression with a binary outcome (1 if the pair belong to the same cluster, 0 otherwise). The exponentiated regression parameters can be interpreted as the adjusted odds ratio of belonging to a same cluster. The second is a regression with a negative binomial likelihood and a logit link, and we model counts (the number of small nucleotide polymorphisms by which the two sequences differ). We present the exponentiated regression parameter as a rate ratio (similar to a Poisson regression analysis), which can be interpreted as the percentage increase in expected count for a 1-unit change in the predictor.

Note that because the unit of analysis is pairs, there are 105 observations (not 15) for the smallest transmission network. Also note that we only model 6 predictor variables in the analysis on the smallest network.

Revised Methods:

“We used a dyadic regression model to understand the factors associated with pairs of individuals (1) belonging to the same transmission network and (2) having more closely related *Mtb* isolates (conditional on belonging to the same network). To answer the first question, we fitted a single logistic regression model using a binary outcome of whether two individuals were in the same network, among individuals who belonged to a network of any size. We included several predictors (age, sex, HIV status, HIV clinic, tuberculosis clinic), and we included the distance between homes as two effects in the model; continuous distance between cases that were close together (< 6 km) and cases that were further apart (≥ 6 km). We chose to model distance in this way because we believe the association between distance and cluster membership may be stronger at shorter distances; we performed model comparisons using AIC, and found that two effects outperformed a single linear effect, and a 6 km threshold outperformed other choices of threshold.

“To answer the second question (i.e., which factors are associated with two individuals in the same network having more similar strain sequences), we performed a separate analysis in which we fitted a negative binomial regression model with a logit link for each transmission network with at least 10 cases (i.e., $\left( \begin{matrix} 10 \\ 2 \end{matrix} \right)=$ 45 pairs), and we modeled the number of SNPs by which pairs differed. This allowed us to investigate predictors of genetic similarity of isolates. We used the same set of covariates as above, and also included the zone in which the individuals resided. We excluded predictors if there was missingness among any individuals within the network or if there were fewer than four pairs of individuals in any level of the predictor.

“We implemented these models using the R package GenePair^(40)^. Regression models with dyadic outcomes will produce effect estimates with erroneously reduced uncertainty if the correlation between dyadic outcomes is not considered^(40, 41)^. GenePair addresses this potential problem by including spatially structured individual-level random effect parameters that induce correlation between the dependent variables^(40)^. For all analyses, we based inference on samples from the joint posterior distribution, removing 10,000 iterations of burn-in and thinning the remaining 25,000 by a factor of 5 to reduce correlation in the posterior samples.”

Revised Results:

“We performed a logistic regression to estimate factors associated with belonging to the same transmission network. We analyzed individuals who belonged to a putative transmission network and had available home location data (n = 389)… We also performed a negative binomial regression to understand factors associated with closer genetic relatedness of isolate strains.”

-       For the transmission flows analysis, please describe whether population size of Zones could affect the flow and how this was accounted for in the analysis and/or the interpretation of the results.

We have changed the methodology by which we calculate the transmission flows. Previously, we had used a model called PhyloFlows, but we have switched to a method which estimates flows directly from the TransPhylo outputs. The results from this new method (y axis) are consistent with results from PhyloFlows (x axis).

We account for the fraction of sequenced strains among individuals with positive cultures in each zone. We note that though these zones differ in population size, the fraction of sequenced strains is relatively stable across zones (with the exception of Zone 3, which had a very small number of individuals with positive cultures). Please see Supplemental Table 2:

| Zone | Population (%) | Cases in Zone | Cases with Sequencing Data (%) |
| --- | --- | --- | --- |
| 1 | 181,021 (20.6%) | 1365 | 188 (14%) |
| 2 | 166,388 (19%) | 860 | 88 (10%) |
| 3 | 9,586 (1.1%) | 55 | 2 (4%) |
| 4 | 124,605 (14.2%) | 1249 | 187 (15%) |
| 5 | 42,500 (4.8%) | 305 | 42 (14%) |
| 6 | 144,702 (16.5%) | 1065 | 125 (12%) |
| 7 | 208,753 (23.8%) | 1678 | 209 (13%) |

-       The first paragraph of Discussion states that the results suggest the need for "action to address the social determinants of tuberculosis. This statement should be better justified. Up to this point in the paper, social determinants are not presented or discussed. Please describe this concept and why the results indicate the need for addressing SDOH.

Thank you for this feedback; we have removed the reference to social determinants of health.

-       The third paragraph of Discussion describes the different characteristics of transmission networks with or without localized foci. It is possible that the clusters in the study represent a snapshot of a distinct stage of an outbreak. Please comment on the extent to which outbreaks at the beginning stages or those that have successfully spread over longer time could explain the patterns observed in this study.

While true that the study necessarily is limited to a distinct snapshot of the epidemic (though we note that this snapshot is approximately 5 years in length), we note that most transmission networks that exhibit a single transmission focus have members that are outside of the focus itself. That is, most of the transmission networks are not strictly limited to a single neighborhood, which can be seen in Figure 4. We agree that it may be possible that these are transmission networks which have recently emerged within a single neighborhood and are in the process of becoming more evenly spread across the city. However, we believe these patterns still must reflect that hyperlocal transmission in these foci is happening, even if these patterns are not expected to persist for these transmission networks indefinitely. Longer term genomic epidemiology studies will be required to understand the ultimate fate of these transmission networks and to determine the degree to which these hotspots of transmission persist through time.

**Reviewer #4:** This spatial and molecular epidemiology analysis of tuberculosis (TB) transmission in Blantyre, Malawi, presents intriguing insights into the patterns of disease spread across and within neighborhoods. The study team was able to successfully sequence and analyze 707 of 3856 culture positive specimens obtained during the study period (2015-2019).
The main findings are that half of the individuals were connected to another case and that two-thirds of transmissions occurred across and not within neighborhoods are important. These results suggest that TB transmission is occurring outside of people's home and immediate vicinity. These findings are important as they highlight that targeting high TB burden neighborhoods for 'door to door' approaches to active TB case finding may have limited value, emphasizing the need for a better understanding of community loci of transmission in high burden settings.

Questions: 
1.      No mention is made of the proportion of inter-household transmission, despite the fact that this could have been inferred from the home GPS coordinates. Could the authors comment on proportions of inter-household TB transmission detected by the study? If none was detected, do the authors think this is an artifact of the low sampling fraction?

Thank you for this comment. We have added a new paragraph investigating household transmission. Reviewer 1 raised a similar question. Please also see our response to Reviewer 1:

We did not have information about whether individuals in our study lived in the same household. This is because it is extremely challenging in a city of ~800,000 people to correctly classify household membership where there is no municipal address system. However, we did attempt to investigate potential household transmission events in two ways. First, we identified individuals who reported having a household contact with TB. Second, we identified plausible shared households based on the proximity of home GPS coordinates. We did not find genomic evidence of transmission for individuals in either group, though we note that this may be related to the coverage of whole genome sequencing. We have added a new paragraph to the result describing these findings:

“We also attempted to identify household transmission events. In total, 270 individuals (2.2% of the TB notifications over the study period) reported a household contact with TB. The final sample dataset included 44 of these individuals (6.1% of the dataset); among these individuals, 21 (47.7%) belonged to a transmission network. Individuals who reported having a household contact with TB were not more likely to belong to a transmission network than individuals who did not report a household contact (two-sample proportion test, p = 0.36). In addition, while 19 individuals could plausibly belong to the same household as another individual in the final sample dataset (i.e. home GPS coordinates were within 50 meters), none of these plausible household pairs belonged to the same transmission network.”

2.      No information is given regarding the TB notification rate in Blantyre during the study period. It would be helpful to have a sense of what proportion the 3856 culture positive individuals form relative to all notified cases in order to better assess the sampling frame.

The first paragraph of the Results section reports the average notification rate over the study period, as well as the minimum and maximum annual notification rates. Previously we had only reported the notification rate in the population of individuals 15 and older. We now report notifications in the total population.

“Between January 2015 and December 2019, 12,238 tuberculosis disease episodes were notified and recorded in ePAL. The mean tuberculosis notification rate over the study period was 279 per 100,000 population per year, which declined from 299 per 100,000 in 2015 to 225 per 100,000 in 2019.”

3.      Could the authors elaborate on the standard diagnostic algorithm employed for TB diagnosis in Malawi during the study period, specifically whether how/where smear microscopy, Xpert testing, and culture are utilized? Given the resource constraints typically associated with TB diagnosis, it is surprising that culture would be routinely obtained in all individuals suspected of having TB. Can you clarify if culture as a first-line test is the standard care, and if not, what criteria are used to select individuals for culture? For example: is culture performed only on individuals who test negative with Xpert or those suspected of having drug-resistant TB? The rationale for selecting individuals for culture is crucial, as it may influence the study's results and the sampling fraction.

In clinical practice, culture is very rarely used to diagnose TB in Blantyre, and was only done by the routine health system where drug resistant TB was suspected (and due to resource limitations in the national programme, often may not have been done). However, for research and surveillance purposes, we collected additional data from all participants at the point of TB notification, including an additional study sputum sample which was tested by both MGIT and smear in the TB research laboratory. While not all patients can produce a sputum sample (and some will have other reasons for non-production), the intention was to collect and culture sputum from all. We performed whole genome sequencing exclusively on bacterial isolates from samples collected for research purposes.

We have updated the Results section to include the number of individuals for whom we had sputum samples available for culture (n = 8,386), and the fraction of those individuals with positive cultures (45.6%).

4.      Figure 3: represents the time-resolved phylogenies with taxa linked to case home location and colored by main lineage. However, the color does not appear in the figure. All dots in figures 3A, 3b and 3C are the same color, and it is unclear which linage the color is meant to represent.

Thank you for noticing this error. All the trees belong to Lineage 4. A previous version of the figure included a lineage 3 tree, and the text about color was left in the legend in error. We have removed it.

5.      Could the authors consider clarifying Figure 6 to enhance the visualization of the data? While the attempt to spatially represent the data is commendable, the figure is difficult to understand. A map of zones colored according to a gradient of intra vs extra zone transmission would be more visually instructive. Another option would be a bipartite graph illustrating intra and cross zone transmission.

Thank you for this suggestion. We have added a grid visualization of the flow estimates to Figure 6:
