# Supplementary material for: Distribution and transmission of M. tuberculosis in a high-HIV prevalence city in Malawi: A genomic and spatial analysis
Source: PLOS Glob Public Health. 2025 Apr 2;5(4):e0004040. doi: 10.1371/journal.pgph.0004040 (PMC11964229; doi:10.1371/journal.pgph.0004040)
Supplement: Attachment — Submitted filename: Response Letter_FINAL.docx [file pgph.0004040.s016.docx]

**Reviewer #2**

Thank you for the opportunity to review the manuscript “Distribution and transmission of M. tuberculosis in a high-HIV prevalence city in Malawi: a genomic and spatial analysis”. I have reviewed a revised version of this manuscript and, as such, have had access to the feedback from previous reviewers and the responses from the authors.

This study leverages high-resolution surveillance data and whole genome sequences; and employs sophisticated spatial and transmission modelling methods to describe the patterns of TB transmission in Blantyre (a city with high burden of TB and HIV-associated TB).

The authors demonstrate that TB transmission in Blantyre is predominantly widespread throughout the city, but this pattern overlaps with multi-focal pockets of hyper-local transmission. These findings potentially translate into policy with the authors proposing that TB control efforts should primarily be applied city wide, rather than spatially-targeted.

I applaud the authors for their considerable efforts. However, I feel there are some major weaknesses with the study and some opportunities for potential revision.

General weaknesses:

These have been identified by previous reviewers and are partially addressed by the authors.

1) Attrition.
Only 6% of the notified cased during the study period were ultimately included in the transmission analysis. Although many of the cases not included represent culture-negative TB, only 18% of culture-positive case could be included in the analysis. This hugely threatens the validity of the transmission inferences the authors make (as these methods assume dense sampling of outbreaks) and the extension of these into TB control recommendations. For instance, it may be that many of the unclustered isolates (46% of sequenced isolates) were only unclustered because the cases within their transmission networks were upsampled. In the extreme (although this is an unlikely scenario), it may be that there are in fact multiple spatially-conserved transmission clusters that were undetected as they were upsampled. Were these hypothetical clusters detected, the conclusion may have been the opposite, and the authors may have recommended spatially-targeted active case findings for primary TB control efforts. Ultimately, while the findings of the study align with our working hypothesis about the nature of TB transmission in high burden settings (that it is widespread), the data it draws its conclusions from are too weak for the study to be confirmatory and it remains hypothesis generating; and its impact is limited.

We have taken several steps to mitigate the potential impact of low sequencing coverage on our findings. First, we allowed for unsampled cases in our inferred transmission networks to account for the uncertainty introduced by low sequencing coverage. We had previously included this as a point in the Discussion section. Second, we focused the analysis on large clusters, which provides some safeguard against the misclassification of truly networked cases as having no known network. We have added this point to the Discussion section. We have also included additional text that more explicitly discusses the potential for misclassification.

“An important limitation of this study is the relatively low fraction of sequencing (18% of culture-positive isolates over the study period). Diagnostic isolates were available for approximately one-third of eligible cases, and lab contamination further reduced the number of sequenced isolates. This poses a challenge for inferring transmission networks, as most existing methods have been designed for more densely sampled outbreaks, **and it is likely that sequenced isolates that were not within transmission networks in our analysis may actually have been linked with other isolates that were just not available or successfully sequenced. We address this limitation using two approaches: first,** we accounted for this by allowing for a higher number of unsampled intermediate cases in transmission networks which introduced additional uncertainty to our transmission inference **as expected. Second, we focused our analysis only on the largest transmission clusters which reduces the sensitivity of our analysis to the expected misclassification of isolates in small (i.e. 2-3 cases) transmission networks as unique isolates.”**

2) Home addresses
The analysis is limited by the reliance on home addresses only for the mapping of spatial foci of transmission, incorporation of spatial correlation into regression models, the use of spatial covariates in the models assessing predictors being in a transmission clusters/genetic relatedness and the analysis of transmission flow across districts. Indeed, the authors already allude to the small role household transmission likely plays in this setting, emphasising the importance of transmission in areas other than home addresses. It is unfortunate that no data on mobility patterns, workplace addresses etc were not available.

We agree that spatial analyses are often limited by the reliance on home location data. In this study, we included additional spatial variables (home zone, TB diagnostic clinic, HIV care clinic) where possible in an attempt to better capture the spaces where individuals may potentially transmit TB. While we are unable to make venue-specific recommendations, we believe this study provides important insights for TB control. Our findings underscore the relatively low contribution of households to overall TB transmission, suggesting that targeted enhanced case finding activities to ‘high risk’ neighborhoods may have limited utility in this setting.

Points for revision:

1) Was any ascertainment bias correction performed for the phylogenies, particularly for the BEAST components, given the inputs were multi-sequence alignments of SNPs rather than whole genomes? If not, the branch lengths and dates for the branching of timed trees may be incorrect. Providing the BEAST input XML file may demonstrate this in addition to improving the reproducibility of the analysis.

Yes, we adapted the XML file to include a correction for constant sites when using SNP alignments instead of the full genome (as suggested by the developers of BEAST here: <https://groups.google.com/g/beast-users/c/QfBHMOqImFE>). As such, the branch lengths and dates are scaled correctly. We have now included an XML file from one of the broad clusters in the supplementary as an example for reproducibility.

We have edited text in the Methods section accordingly:

“We used a relaxed lognormal substitution rate and constant coalescent population tree prior, along with adapting the XML file to include a correction for invariant sites.”

2) The authors demonstrate the sharing a TB clinic is associated with a higher odds of a pair of cases being in the same transmission network; and (for network #2) that this is associated with a higher risk that a pair of cases are closer genetically related. I am unsure of what to make of this. Perhaps the authors can expand the rationale for including this is a covariate and providing some interpretation of its significance. For example, is this intended as a proxy for people sharing the same geographic region (if so, is this colinear with distance between home addresses)? Or do the authors think it is causally related to being in a transmission network in some other way? Does this finding have implications for TB control?

Clinics represent both a potential site of transmission (particularly when TB services are nested in a primary healthcare clinic) and a community level effect (which may be not captured in the larger ‘zone’ effect or in the continuous distance effect).

We have made several changes to the text to clarify why we included clinics and what the implication of a clinic effect may be for TB control. First, we would like to clarify that ‘TB Clinic’ and ‘HIV Clinic’ are shorthand for identifying a clinic where an individual received care for either of these diseases. We have clarified this within the text where necessary. Second, we have added new text in the Discussion section which interprets this finding and discusses implications for TB control measures:

“However, we found that receiving a TB diagnosis at the same clinic was associated with a greater odds of belonging to the same transmission network, independent of home location of individuals. While this could be explained by transmission within healthcare settings, it could also reflect a shared neighborhood effect that was not completely captured by home location since people living in the same neighborhood would be more likely to attend the same clinic.”

With regards to concerns about co-linearity, we note that approximately 12% of pairs living within 6 km of each other attended the same TB. We found there was a very weak correlation between household distance (up to 6 km) and being diagnosed at the same clinic (Pearson’s correlation coefficient = 0.003, p = 0.05) or receiving HIV-related care at the same clinic (-0.006, p = 0.005).

3) For the regression analysis investigating the outcome of pairs of cases belonging to the same transmission network: What is the rationale for only including clustered cases (n=389) rather than all cases (707)? Does this potentially bias these results? For example what if many of the unclustered cases share the same TB clinics as those in putative transmission networks and their exclusion inflated the effect size of the association between sharing a TB clinic and belonging to the same transmission network?

We initially included only pairs of cases in a transmission network to reduce the overall complexity and run time of the model. GenePair includes a random effect for each individual included the analysis, and the model can quickly become prohibitively large. We have reperformed the analysis, now including all individuals. These results of this new analysis are similar to our original findings.

We have removed the sentence in the Method section indicating the exclusion of cases that were not part of a transmission network. We have updated the Results section to accordingly:

“We analyzed individuals who had **both WGS data and home location data (n = 707)**, and found that two individuals had a higher odds of belonging to the same putative transmission network if they shared a tuberculosis diagnostic clinic (adjust odds ratio (aOR) 1.61 95% highest posterior density interval (HPD) **1.16**, 2.13) or were both male (**aOR 1.48, 95% HPD 1.02, 2.03**) (Figure 5A). A 1 km increase in the distance between home location was associated with a **0.83 (0.78, 0.88)** aOR of belonging to the same cluster for distances up to 6 km. Each 1 km increase in distance above the 6 km threshold was associated with a 0.91 (**0.89, 0.93**) aOR. We did not find a significant association between shared HIV clinic **exposure (aOR 0.86, 95% HPD 0.58, 1.21), or HIV status (aOR for HIV positive pairs: 0.94 [0.66, 1.30]; aOR for HIV negative pairs: 1.37 [0.93, 1.92])** and belonging to the same putative transmission network.”

4) In Figure 5B, there are no lines for the estimates for same TB clinic and same HIV clinic for network #43

We exclude covariates when there is an insufficient number of pairs to make inference about effects; we judge this to occur when there are fewer than four pairs (three or fewer individuals) in a given covariate level. We note this both in the Methods section and in the figure legend.

5) The data availability statement does not currently specify any publicly available deposition. While it does not appear to be mandated by PLOS GPH , the publishing of the analysis code would greatly enhance the reproducibility of the work and offers an opportunity for others to learn from, what is in my opinion the most impactful part of this work, the methods.

Thank you for raising this issue. We do intend to make the whole genome sequencing data public upon acceptance for publication.

Conclusion:
I commend the authors for their substantial efforts in collecting and harmonising multi-modal data sources and for the rigorous application of the methods employed in this study. However, practical challenges related to data availability have significantly constrained the novelty and overall impact of the work. Despite these limitations, the study remains a commendable contribution to the field.

I would like to raise an important issue regarding equity in global health research, particularly given PLOS Global Public Health's mission statement: “PLOS Global Public Health addresses deeply entrenched global inequities in public health...” It was disappointing to note that only 3 out of 18 contributing institutions were Malawian, and just 6 of the 24 authors were affiliated with Malawian institutions, with the majority of representation coming from the Global North. I recognise the complexities of funding, study design, data analysis, and authorship dynamics are unique and cannot be discerned from an author list alone. However, equity should remain a fundamental consideration for global health researchers. Moving beyond a culture of "parachute research" is essential for ensuring meaningful collaboration and representation. I do not intend to be accusatory but rather to emphasise the ongoing relevance of global heath equity going forward. This issue has been extensively discussed both in this journal (e.g., DOI:10.1371/journal.pgph.0000160, DOI:10.1371/journal.pgph.0003141) and elsewhere (DOI:10.1016/S0140-6736(24)02323-7).

We thank the reviewer for this important comment, and agree with the point they are making. These are issues that we have long grappled with. In response, we note that the data that were analysed in this manuscript came from a long-established research partnership between the Malawi-Liverpool-Wellcome Programme, The Blantyre District Health Office, and the Kamuzu University of Health Sciences. Enhanced surveillance of tuberculosis notifications across the city of Blantyre commenced in 2009, and has been ongoing since, supported by extensive capacity development, north-south, and south-north knowledge exchange, and training and development – evidenced by the large numbers of Malawian-led publications and scientists that have progressed to local and international leadership positions over this 16 year period. Although in this manuscript 3 authors were Malawian, many of the “northern” researchers are long-term residents and were living and working in Malawi since 2009. Until now however, there has been extremely limited capacity for whole genome sequencing analysis of TB in Malawi. Indeed, this is the first collaborative project that set out to undertaking TB epidemiology analysis using these datasets, and necessarily required expertise in sequencing, modelling, and computational methods that have hitherto not been available in Malawi. There has been considerable capacity development through this project, and we anticipate that future studies will be led by Malawian team members.

**Reviewer #3**

Chitwood and colleagues have conducted an incredibly sophisticated analysis of a complex problem. Using routinely collected electronic health data and whole genome sequencing, they have conducted an insightful analysis of TB transmission in Blantyre, with findings that are relevant to policymakers. I note the prior comments from four peer-reviewers via another journal have already been answered adequately. The manuscript is well-written and conclusions are not overstated. I have the following additional suggestions/requests for clarification which are largely minor:

1. Suggest make it clear in methods that the only locations being considered for the spatial analysis were the household, the TB clinic or the HIV clinic. This does also come through clearly in the discussion section.

We have updated the text in two Methods subsections to better reflect the use home location in spatial analyses.

Identification of spatial foci of transmission

“For each putative transmission network with at least five cases, we investigated spatial areas where individuals with tuberculosis had relatively higher likelihoods of transmission to or from others in the area**, based on individuals’ reported home locations**.”

Transmission flow analysis

**We mapped a sequenced TB case to a zone based on their reported home location.**

2a. Suggest make it clear in the figures (e.g. figure 1A/B) which location is being referred to e.g. that the location being highlighted is the household (if I understood this correctly) rather than the location of their clinic?

2b. For Figure 1B, is this HIV prevalence only with regards to the TB cases in dataset, or Blantyre-wide estimates independent of the study? Suggest make clear in the figure's title.

We have updated the figure legend to indicate that maps are based on reported home locations and to clarify that the map of HIV prevalence estimates are city-wide.

“Map of Blantyre, Malawi with (**A**) culture positive tuberculosis case notifications per 100,000 population per year over the study period; (**B**) HIV prevalence **estimates for the city population (with and without TB-coinfection), aged 15-49 years**; (**C**) all notified tuberculosis cases, colored by major lineage or shown in grey if no sequencing data were available; (**D**) inset map indicating the location of Blantyre in Malawi. **All points indicate reported home locations.** Points have been jittered for privacy.”

3. Within the results section "Whole genome sequencing analysis", suggest include denominators with the various numerators so that the percentages are clear to the reader. For example, to reflect on the fact that only estimated 8% of all those who could have potentially been included in study dataset landed up having matched sequencing and clinical/epi data.

We’d like to clarify that whole genome sequencing analyses are limited to individuals with positive TB cultures. As such, our study dataset includes 18% of those who could have potentially been included. We have updated this subsection to clarify denominators and to include the percentage of both isolates available for sequencing and positive TB cultures.

“Among sequenced isolates, 861 (**64.6% of available isolates, 22.3% of positive cultures**) passed quality control checks and 717 (**53.8% of available isolates, 18.6% of positive cultures**) were found to be pure (non-mixed) samples, which were included in the final sample dataset.”

4a. I appreciate the addition of table 1 based on prior peer-review. Does the available data allow for an additional column of all Blantyre TB cases, to compare between the overall culture-positive TB cases and the ones included in this study?
4b. Furthermore, in line with the main conclusions of the study, could additional rows be added with regards to the presence of a household contact, and with regards to zone inhabited, to know whether either of these were under- or over-represented in their study sample?

We agree that it would be useful for the reader to compare all TB cases to culture-positive cases and sequenced cases. We have included these details as a new supplemental table (Supplemental Table 3). In addition, approximately 60% of culture-positive TB cases declined to report known TB contacts and approximately 50% did not report a home location. We feel it would create confusion if we included these factors in the main text table. We have included these in the supplemental table:

|  | **All TB Cases**  N = 12,238 | **All Culture-Positive**  N = 3,865 | **Final Sample dataset**  N = 717 |
| --- | --- | --- | --- |
| Sex |  |  |  |
| Male | 7,438 (61%) | 2,542 (66%) | 496 (69%) |
| Female | 4,800 (39%) | 1,323 (34%) | 221 (31%) |
| Age | 35 (26, 43) | 34 (27, 40) | 33 (26, 39) |
| HIV status |  |  |  |
| Positive, on ART | 7,146 (58%) | 2,043 (53%) | 380 (53%) |
| Positive, Not on ART | 714 (5.8%) | 244 (6.3%) | 50 (7.0%) |
| Negative | 4,073 (33%) | 1,489 (39%) | 269 (38%) |
| Not Collected | 305 (2.5%) | 89 (2.3%) | 18 (2.5%) |
| Patient Type |  |  |  |
| New | 10,608 (87%) | 3,401 (88%) | 647 (90%) |
| Relapse | 1,232 (10%) | 373 (9.7%) | 61 (8.5%) |
| Other | 398 (3.3%) | 91 (2.4%) | 9 (1.3%) |
| Known Contact |  |  |  |
| Yes | 659 (5.4%) | 269 (7.0%) | 86 (12%) |
| No | 4,479 (37%) | 1,668 (43%) | 564 (79%) |
| Missing | 7,100 (58%) | 1,928 (50%) | 67 (9.3%) |
| City Zone |  |  |  |
| 1 | 1,228 (10%) | 490 (13%) | 156 (22%) |
| 2 | 760 (6.2%) | 247 (6.4%) | 73 (10%) |
| 3 | 49 (0.4%) | 11 (0.3%) | 2 (0.3%) |
| 4 | 1,100 (9.0%) | 446 (12%) | 161 (22%) |
| 5 | 272 (2.2%) | 103 (2.7%) | 35 (4.9%) |
| 6 | 895 (7.3%) | 376 (9.7%) | 103 (14%) |
| 7 | 1,422 (12%) | 545 (14%) | 173 (24%) |
| Missing | 6,512 (53%) | 1,647 (43%) | 14 (2.0%) |
| n (%); Median (Q1, Q3) | | | |

5a. My understanding is that clinic sputum culture samples were supplemented by research tests. In response to a reviewer's comments, it was noted that sequencing was performed on the research tests. Suggest include context of how samples were collected or how they fit within the algorithm of routine testing in Malawi.
5b. With this, could the authors provide context on whether all included samples were sourced from passive case finding and individuals self-presenting to their TB clinics? And thereby a pool of infectious (possibly asymptomatic) TB disease is thereby not considered in this dataset?

Thank you for this question. As noted in the methods, participants in this analysis were people notified with TB at clinics throughout the city of Blantyre, and thus were identified through passive case finding activities. We have clarified this in the Method section: “This retrospective study included people **identified through passive case detection and** diagnosed with tuberculosis in Blantyre, Malawi between 1 January 2015 and 31 December 2019.”

To clarify testing algorithms, sputum culture is not routinely available through the routine health system for diagnosis of TB in Malawi (only available in very rare/specialist situations). Thus, participants in this analysis were diagnosed by passive case detection in the routine health system on the basis of sputum smear, Xpert, or clinical diagnosis.

However, as part of our enhanced surveillance system, each adult notified with TB (where able) additionally provided a sputum sample for research purposes at the point of notification that underwent culture and smear in the joint MLW-KUHeS TB Research laboratory. Results of this culture were reported back to clinicians and patient where relevant, and to the national TB programme for surveillance, monitoring and evaluation. It is from these research culture specimens that sequencing activities were undertaken.

Of course, as in any high TB burden setting where case detection is sub-optimal, there will be a people who are undetected by passive case finding activities. As previously noted, we have taken several steps to address missingness in our genomic analyses. We do note however, that we have undertaken two large active case finding trials in Blantyre (<https://doi.org/10.1093/cid/ciad238>; https://doi.org/10.1371/journal.pgph.0002683), and rapidly declining case notification rates and prevalence suggests that the case detection rate is likely to be higher than comparable settings.

6. Finally, I also think there is a small typo in this sentence from methods section "These data were combined in a Bayesian model to derive*d* highly spatially resolved HIV prevalence estimates, as described previously"

Thank you for identifying this typo; we have fixed it.

**Additional Editor Comments (if provided)**

I would like to see a more detailed discussion of the study limitations, given the feedback by the reviewers.

We have included substantial new text in the Discussion about limitations related to the limited type of spatial data as well as low sequencing coverage.
